# Differential Proteomic Profiles of *Pleurotus ostreatus* in Response to Lignocellulosic Components Provide Insights into Divergent Adaptive Mechanisms

**DOI:** 10.3389/fmicb.2017.00480

**Published:** 2017-03-23

**Authors:** Qiuyun Xiao, Fuying Ma, Yan Li, Hongbo Yu, Chengyun Li, Xiaoyu Zhang

**Affiliations:** ^1^Key Laboratory of Molecular Biophysics of MOE, College of Life Science and Technology, Huazhong University of Science and TechnologyWuhan, China; ^2^Key Laboratory of Agro-Biodiversity and Pest Management of Education Ministry of China, Yunnan Agricultural UniversityKunming, China

**Keywords:** *Pleurotus ostreatus*, proteomics, white-rot fungus, fungal adaptability, lignocellulose

## Abstract

*Pleurotus ostreatus* is a white rot fungus that grows on lignocellulosic biomass by metabolizing the main constituents. Extracellular enzymes play a key role in this process. During the hydrolysis of lignocellulose, potentially toxic molecules are released from lignin, and the molecules are derived from hemicellulose or cellulose that trigger various responses in fungus, thereby influencing mycelial growth. In order to characterize the mechanism underlying the response of *P. ostreatus* to lignin, we conducted a comparative proteomic analysis of *P. ostreatus* grown on different lignocellulose substrates. In this work, the mycelium proteome of *P. ostreatus* grown in liquid minimal medium with lignin, xylan, and carboxymethyl cellulose (CMC) was analyzed using the complementary two-dimensional gel electrophoresis (2-DE) approach; 115 proteins were identified, most of which were classified into five types according to their function. Proteins with an antioxidant function that play a role in the stress response were upregulated in response to lignin. Most proteins involving in carbohydrate and energy metabolism were less abundant in lignin. Xylan and CMC may enhanced the process of carbohydrate metabolism by regulating the level of expression of various carbohydrate metabolism-related proteins. The change of protein expression level was related to the adaptability of *P. ostreatus* to lignocellulose. These findings provide novel insights into the mechanisms underlying the response of white-rot fungus to lignocellulose.

## Introduction

To adapt to changing environments, fungi have developed mechanisms to sense and respond to a multitude of environmental factors such as different carbon sources (Akai, 2012; Kües, 2015). *P. ostreatus* is a white-rot fungus that can be easily cultivated on a variety of lignocellulosic substrates, owing to its ability to degrade cellulose, lignin, and hemicellulose through the action of complex oxidative and hydrolytic enzymatic systems (Fernández-Fueyo et al., 2016). However, lignin does not act as the sole source of carbon and energy; the degradation of lignin by white-rot fungi enables access to holocellulose, which is the carbon and energy source for this species. Presumably, cellulose and hemicellulose provide carbon and energy sources for growth, whereas lignin serves a barrier to prevent *P. ostreatus* from attacking polysaccharides. Lignin likely acts as the target for enzymes participating in degradation. manganese peroxidase (MnP) and laccase are the major oxidative enzymes secreted by *P. ostreatus* that are responsible for the oxidation of lignin and a wide range of lignin-analogous compounds (Wan and Li, 2012). In addition, various auxiliary enzymes generate hydrogen peroxide, which is required for oxidation of lignin. During the lignin degradation process, aromatic radicals are produced that catalyze subsequent degradation, generating potentially toxic molecules that trigger a defense response to protect the fungus from harmful environments (Li et al., 2015b). Primary mycelial enzymes play important roles in cellular processes involving utilization of lignocellulose; earlier studies revealed that the use of conditional transitions in biological pretreatment would affect the expression of the white rot fungi genes encoding ligninolytic enzymes at the transcriptional level (Sindhu et al., 2016).

After the lignin barrier is broken, *P. ostreatus* attacks lignocellulosic polysaccharides. The most abundant hemicellulose is xylan, which is composed of pentoses such as xylose, whereas the most abundant form of cellulose is glucose. The degradation of hemicellulose and cellulose is dependent on carbohydrate-active enzymes, whose functions do not overlap (Lombard et al., 2014); therefore, a large number of different enzymes is required for hemicellulose and cellulose degradation.

Flavin adenine dinucleotide (FAD)-dependent proteins are a current research focus, as these enzymes play important roles in lignocellulose oxidation (Levasseur et al., 2013). Flavin-mediated oxidation, which involves dioxygen as the electron acceptor, is thermodynamically favorable (Hamdane et al., 2015). Previous studies of the response of flavoproteins to lignin have focused on the role of extracellular flavoprotein during lignocellulose degradation (Hernández-Ortega et al., 2012); however, there have been few reports on the role of intracellular flavoproteins in lignocellulose degradation.

In addition, the molecular mechanisms underlying the mycelial response to hemicellulose, cellulose, and lignin remain poorly understood. Recent studies have shown that cellular responses to lignin derivatives are critical for optimization of ligninolytic conditions in fungal cells (Simon et al., 2014). Therefore, elucidation of the catalytic functions of lignin-responsive enzymes is necessary.

The degradation of lignocellulose by *P. ostreatus* plays a role in the acclimation of this fungus to the environment. Adaptation to the specific environment is mediated via profound changes in the expression of genes, which leads to changes in the composition of the fungal transcriptome, proteome, and metabolome (Gaskell et al., 2016). On the basis of their activity, proteins are traditionally classified as catalysts, signaling molecules, or building blocks in cells and microorganisms. Therefore, researchers have attempted to explore the mechanism underlying the interaction between fungi and lignocellulose by proteomics. Proteomics analysis of the filamentous fungus *Trichoderma atroviride* grown on cell walls identified 24 upregulated proteins, including fungal cell wall-degrading enzymes such as *N*-acetyl-β-d-glucosaminidase and the 42-kDa protein endochitinase (Grinyer et al., 2005). Proteomic analysis of *Botrytis cinerea* revealed that proteins such as malate dehydrogenase or peptidyl-prolyl cis–trans isomerase from the mycelium were differentially expressed among strains when using CMC as the sole carbon source; these proteins are involving in host-tissue invasion, pathogenicity, and fungal development (González-Fernández et al., 2014). These studies attempted to elucidate the effects of plant cell wall composition on microbes by mixing lignocellulose or cellulose as substrates; however, they only provide limited evidence that the main components of the plant cell wall alter the gene expression in fungal cells, and that lignin and hemicellulose might also affect the growth and protein expression of fungal cells. To date, few studies have been published regarding the intracellular proteomics of the white-rot fungal response to lignocellulose.

In this work, we performed two-dimensional protein fractionation coupled with mass spectrometry to analyze the potential biological differences among *P. ostreatus* cells grown on different lignocellulose media. *P. ostreatus* was grown in Kirk's medium to which lignin, xylan, and CMC were added; this medium is commonly used in studies of the response of white-rot fungus to lignocellulose. We compared the biomass and FAD concentration in cells during cultivation. Next, proteomic profiles of *P. ostreatus* under lignocellulose culture conditions were obtained. The 2-DE expression profiles were used to analyze the intracellular proteins differentially expressed in various substrates, and differentially expressed proteins were identified by MALDI-TOF-MS. Finally, the metabolic pathways involving in the lignocellulose response in *P. ostreatus* were examined according to the differentially expressed proteins in the various substrates.

## Materials and methods

### Microorganism and cultivation

*P. ostreatus* isolate BP2 obtained from the Culture Collection Center, Huazhong Agriculture University (Hubei, China) was used in this study. The strain was maintained on potato dextrose agar (PDA) slants at 4°C and activated for 1 week on new PDA slant before use, then transferred into potato dextrose broth (PDB) medium for 7 days at 28°C as inoculum.

In order to exclude influence of other organics, the strain was inoculated into a 250 ml flask with 100 mL modified Kirk's liquid medium which just contain basal salt component as basic medium (Taniguchi et al., 2005). The Kirk's liquid medium contained: 9 × 10^−3^ mol/L KH_2_PO_4_, 3 × 10^−3^ mol/L MgSO_4_.7H_2_O, 2 × 10^−5^ mol/L ammonium tartrate, 3 × 10^−4^ mol /L CaCl_2_.2H_2_O, 5 × 10^−2^ mol/L glucose, and 10 ml/L trace element contained: 7.8 × 10^−3^ mol/L amino acetic acid, 1.2 × 10^−3^ mol/L MgSO_4_.H_2_O, 2.9 × 10^−3^ mol/L MnSO_4_.H_2_O, 1.7 × 10^−2^ mol/L NaCl, 3.59 × 10^−4^ mol/L FeSO_4_.7H_2_O, 7.75 × 10^−4^ mol/L CoCl_2_, 9.0 × 10^−4^ mol/L CaCl_2_, 3.48 × 10^−4^ mol/L ZnSO_4_.7H_2_O, 4 × 10^−5^ mol/L CuSO_4_.5H_2_O, 2.1 × 10^−5^ mol/L AlK(SO_4_)_2_.12H_2_O, 1.6 × 10^−4^ mol/L H_3_BO_3_, 4.1 × 10^−5^ mol/L NaMoO_4_.2H_2_O.or 100 ml Kirk's liquid medium supplemented with 0.5 g lignin(Sigma), xylan(Sigma), or cellulose(Sigma). All experiments were accompanied by controls that lacked the lignocellulose amendment. The mycelia were collected after 7 days incubation in dark at 28°C with continuous stirring at 120 r/min, and the cultures were centrifuged for collection washed with sterilized MilliQ water for several times to separate from medium, then kept at −80°C for use.

### Growth measurement

The mycelial dry weight was used to characterize *P. ostreatus* growth condition. Base on the method reported before (Taniwaki et al., 2006), the mycelium cultured in lignin as mentioned before was weighed after cultured for 0, 3, 5, 7, 9, 11 days. Three individual cultures of the mycelium were weighed at every time point.

### Analysis of FAD concentration during *P. ostreatus* growth

Mycelium proteins were obtained using a dynamic high pressure homogenizing (GEA Niro Soavi S.p.A), and proteins were quantified by BCA method. Intracellular FAD concentration was measured using an FAD Colorimetric/Fluorometric Assay Kit (BioVision). Experimental methods refer to product description.

### Laccase activity assays

Laccase activity was determined spectrophotometrically as previous study described by with 14 μmol of ABTS as the substrate (Srinivasan et al., 1995). All the assays were done at pH 3.0, the optimum pH for laccase of *P. ostreatus* with ABTS as the substrate.

### 2-De analysis of mycelia protein

Frozen mycelia were used to extract total myceliaproteins by the TCA-acetone precipitation method (Rabilloud et al., 2010). Mycelia (dry weight of 1 g) was ground to a fine powder under liquid nitrogen and was collected into 50 ml microcentrifuge tubes. Three individual cultures of the mycelium were harvested and extracted separately. Twenty milliliters cold acetone (−20°C, 10% w/v trichloroacetic acid (TCA), 0.1% w/v dithiothreito (DTT, Bio-Rad), 1 mmol/L phenylmethanesulfonyl fluoride (PMSF, Sigma) was added into the tube. After the samples were resuspended totally, the tube was incubated at −20°C for more than 12 h, and then the samples were centrifuged for 20 min at 14,000 r/min. The resulting pellet was washed with 15 mL cold acetone (0.1% w/v DTT, 1 mmol/L PMSF), then centrifuged at 14,000 r/min for 20 min. This washing procedure was repeated twice and final pellet was resuspended. The pellet was vacuum-dried and solubilized with lysis buffer containing 7 mmol/L urea, 2%CHAPS (Sigma), 10 mmol/L DTT and 0.5% biolytes (Bio-Rad). After fully dissolved, the samples were stored at −80°C for 2-DE analysis. Protein concentration was determined using Bradford's method with bovine serum albumin as standards (Fernández and Novo, 2013). Ready strip IPG strips (18 cm, 4–7 linear pH gradient, Bio-Rad) were rehydrated for 12 h with 800 μg of protein sample as most mycelial proteins were in this range according to previous studies (Jami et al., 2010). Then the IPG were carried out for the first electrophoretic dimension in a Protean IEF-Cell (Bio-Rad). The isoelectric focusing was performed with a limiting current of 50 μA/strip following the program setting: (i) 250 v, rapid, 0.5 h. (ii) 1,000 v, rapid, 0.5 h. (iii) 9,000 v, liner, 4.5 h. (iv) 9,000 v, rapid, 75,000 vh(v) 500 v, rapid, 1 h. The IPG strips were treated twice for at least 30 in with SDS equilibration buffer (6 mmol/L urea, 1.5 mmol/L Tris-Cl with pH 8.8, 30% v/v glycerol, 2% (w/v) SDS, 0.001% bromophenol blue). Ten milligrams per milliliters DTT was add to the equilibration buffer in the first step, and 25 mg/mL iodoacetamide was added in the second step. The second dimensional SDS-polyacrylamide electrophoresis (SDS-PAGE) was performed on v/v 12.5% acrylamide gel (v/v 2% SDS) by using a Protean II xi Cell system (Bio-Rad). Coomassie PAGE Blue (Bio-Rad) was used to stain the gels. The finished gels were scanned with GE Gel Scan system (GE) and analyzed with PDQuest software (7.0.1 version, Bio-Rad). In order to verify the significant change of protein/spot, three replicate 2-DE gels were visually compared by using PDQuest software. The spots/proteins appeared in all three biological replicate could be considered the infallible spots/proteins, Finally, only differences with a ratio lignocellulose/control (R) 0.5 > R or R > 2 (CV < 25%), and with a *t*-test (*p* < 0.05), were considered as significant. The theoretical pIs were calculated using the ExPASy Compute pI/Mw tool (http://web.expasy.org/compute_pi/).

### ESI-MS/MS of 2-De spots

Then, we performed MALDI-TOF/TOF to identify significantly changed spots in one or two cultures compared with that in the control. Spots from 2-DE gels were excised and digested with trypsin for 20 h. The resulting peptide mixtures were desalted using ZipTips C18 (Millipore), and eluted onto a 96-well MALDI target plate. Then, 2 mL samples on the plate were mixed with 1 mL supersaturated CHCA solution with 0.1% TFA and 50%ACN. Mass spectrometric analysis were measured on 5800 MALDI-TOF/TOF (AB SCIEX). Briefly, mass data acquisitions were piloted by 4000 Series Explorer Software v3.0 using batched-processing and automatic switching between MS and MS/MS modes The PMF data were collected and blasted in JGI database using MASCOT software (http://matrixscience.com).

## Results

### Lignocellulose components influence the growth of mycelium

**Figure 1:**
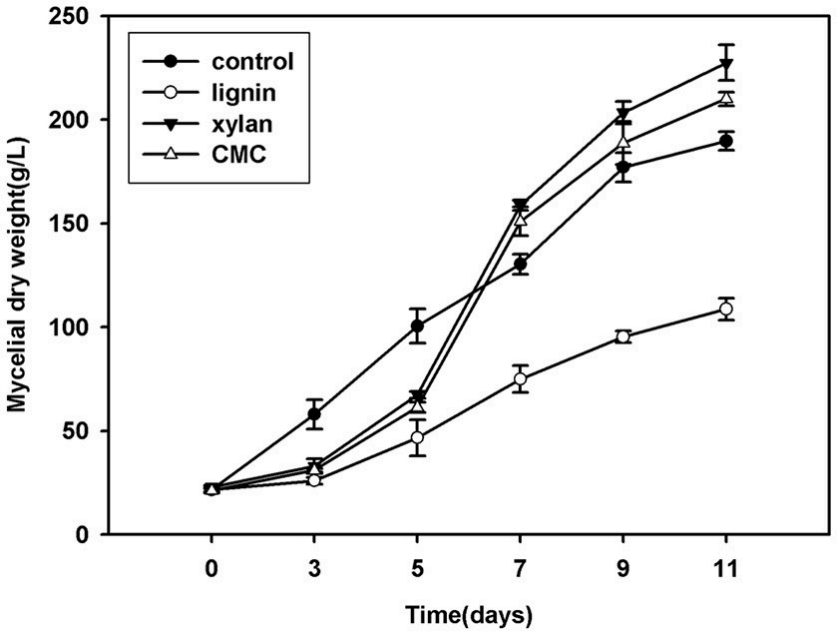
**Growth curve of mycelium (mycelium dry weight) in Krik's, lignin, cellulose, and xylan for 11 days**.

*P. ostreatus* grown in Kirk's medium supplemented with lignin, xylan, and CMC was used to study the relative intensity of proteins affected by lignocellulose, and Krik's medium without lignocellulose was used as control. The mycelial dry weights of colonies grown on lignocellulose significantly differed from those of the control (Figure 1). The growth of fungal mycelium was suppressed on lignin relative to other cultures. Xylan and CMC served as slow-acting carbon resources; accordingly, the biomass of mycelium in xylan and CMC accumulated slowly at first, and then began to surpass that of the control 7 days after inoculation. Compared with the control, lignocellulose supplementation suppressed mycelial growth for the first 7 days of culture; subsequently, mycelia underwent adaptation to xylan and CMC, resulting in rapid growth of *P. ostreatus* in these medium.

### Lignocellulose components influence the FAD levels of mycelia

**Figure 2:**
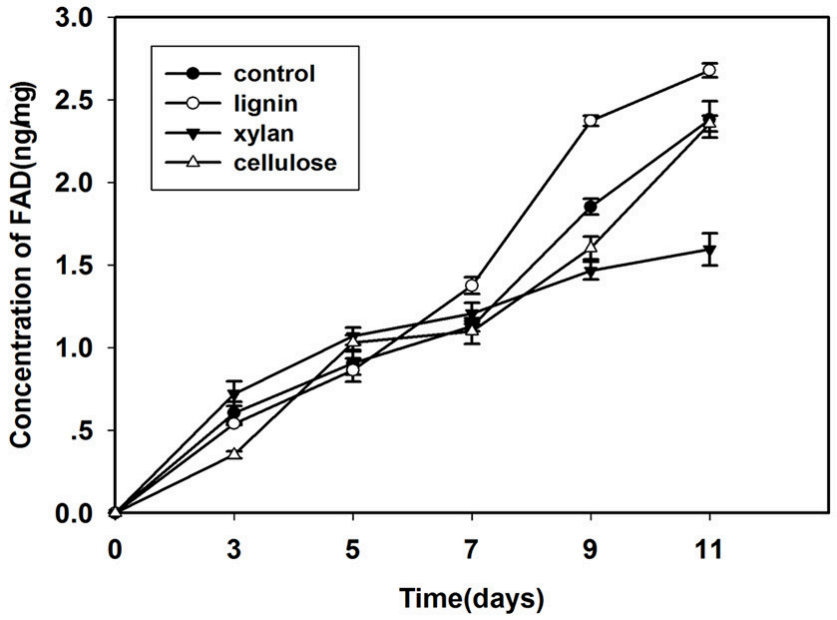
**Concentration of FAD in mycelium that cultured in control, lignin, xylan, and CMC for 11 days**.

FAD is a redox cofactor that plays an important role in metabolism (Figure 2). The primary sources of reduced FAD levels during eukaryotic metabolism are the citric acid cycle and beta oxidation reaction pathways. FAD accumulates with time, especially during growth on lignin. After inoculation for 7 days, the FAD concentration was higher in fungi grown in lignin than in other cultures.

### Lignin influence laccase activity

**Figure 3:**
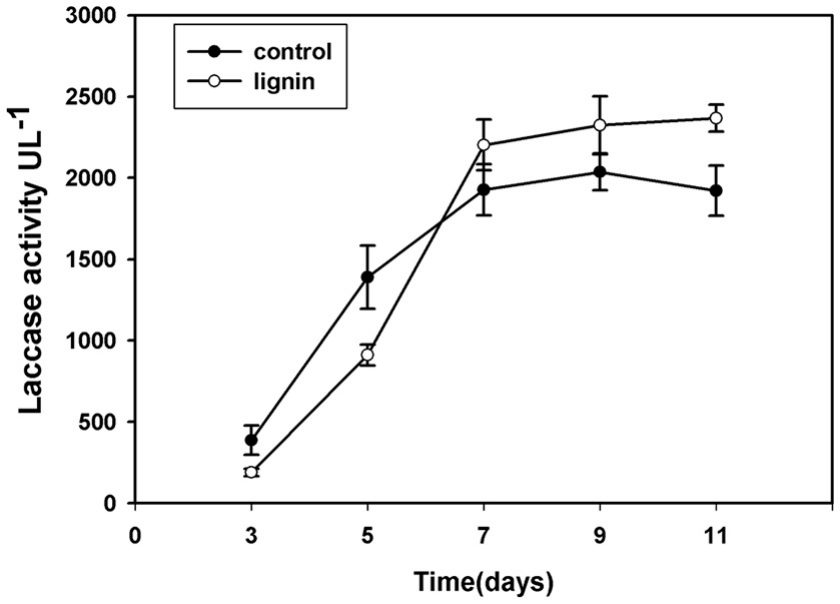
**Changes of laccase activity in Kirk's liquid medium supplemented with lignin and without lignin (control) after inoculation for 11 days**.

Since laccase is the most important extracellular enzyme responsible for lignin modification, we examined its activity in the lignin group (Figure 3). After inoculation, the laccase activity in this group was lower than that in the control for the first 5 days; however, after culturing for 7 days, laccase activity in the lignin group was higher than that in the control.

### Differences between the mycelial proteomes during growth in lignocellulose and in the control medium

**Figure 4:**
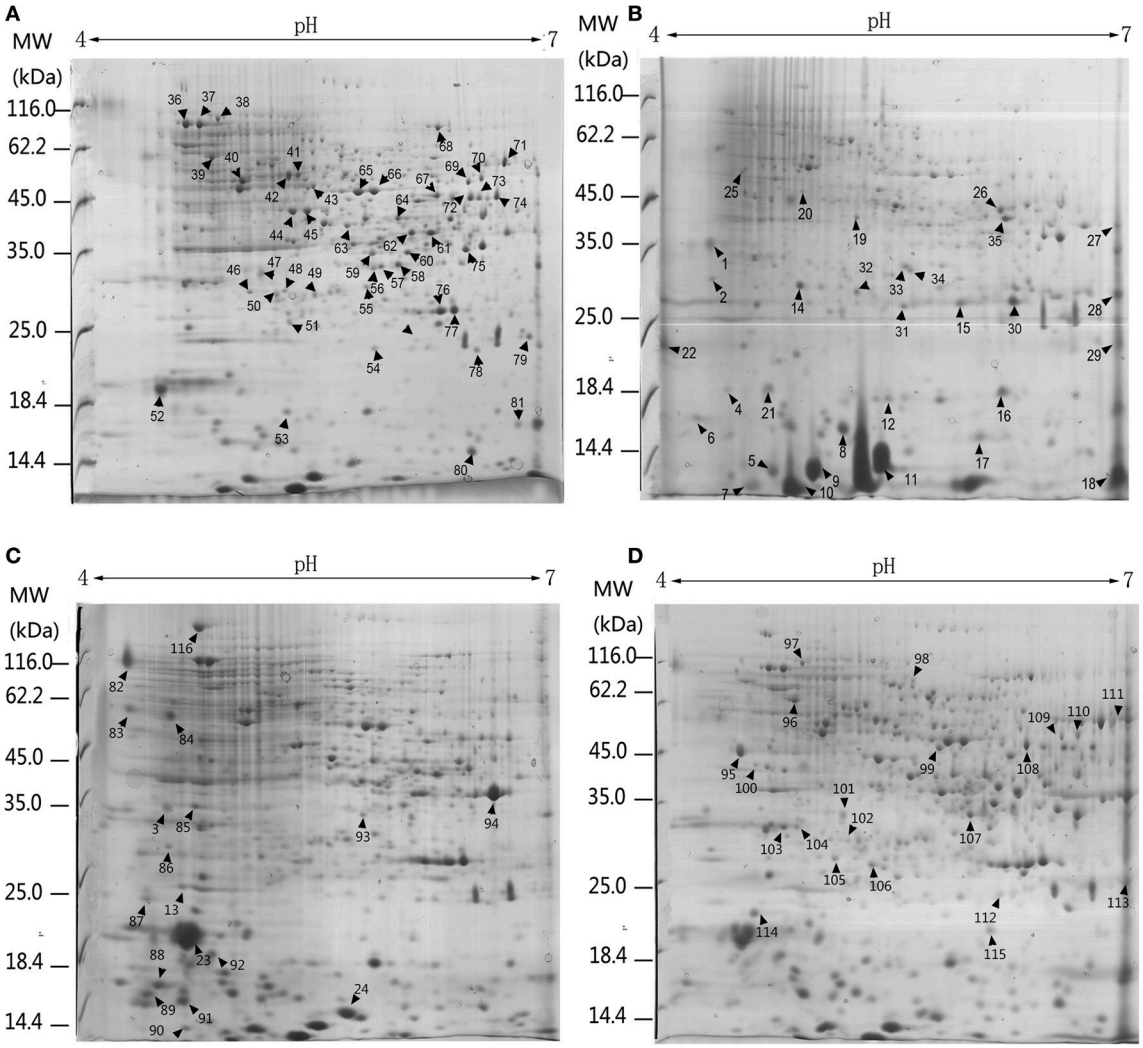
**2-DE analysis of differential expressed proteins in ***P. ostreatus*** grown in liquid substrates supplemented with different components of lignocellulose**. Arrows and numbers refer to differential expressed proteins **(A)** Kirk's liquid medium (control). **(B)** Kirk's liquid medium supplemented with lignin; **(C)** xylan; **(D)** CMC. The pH of the isoelectric focusing gel ranging from 4 to 7 is shown on the top of each gel. Protein ladder (molecular weight, MW) is shown on the left of the gels.

**Figure 5:**
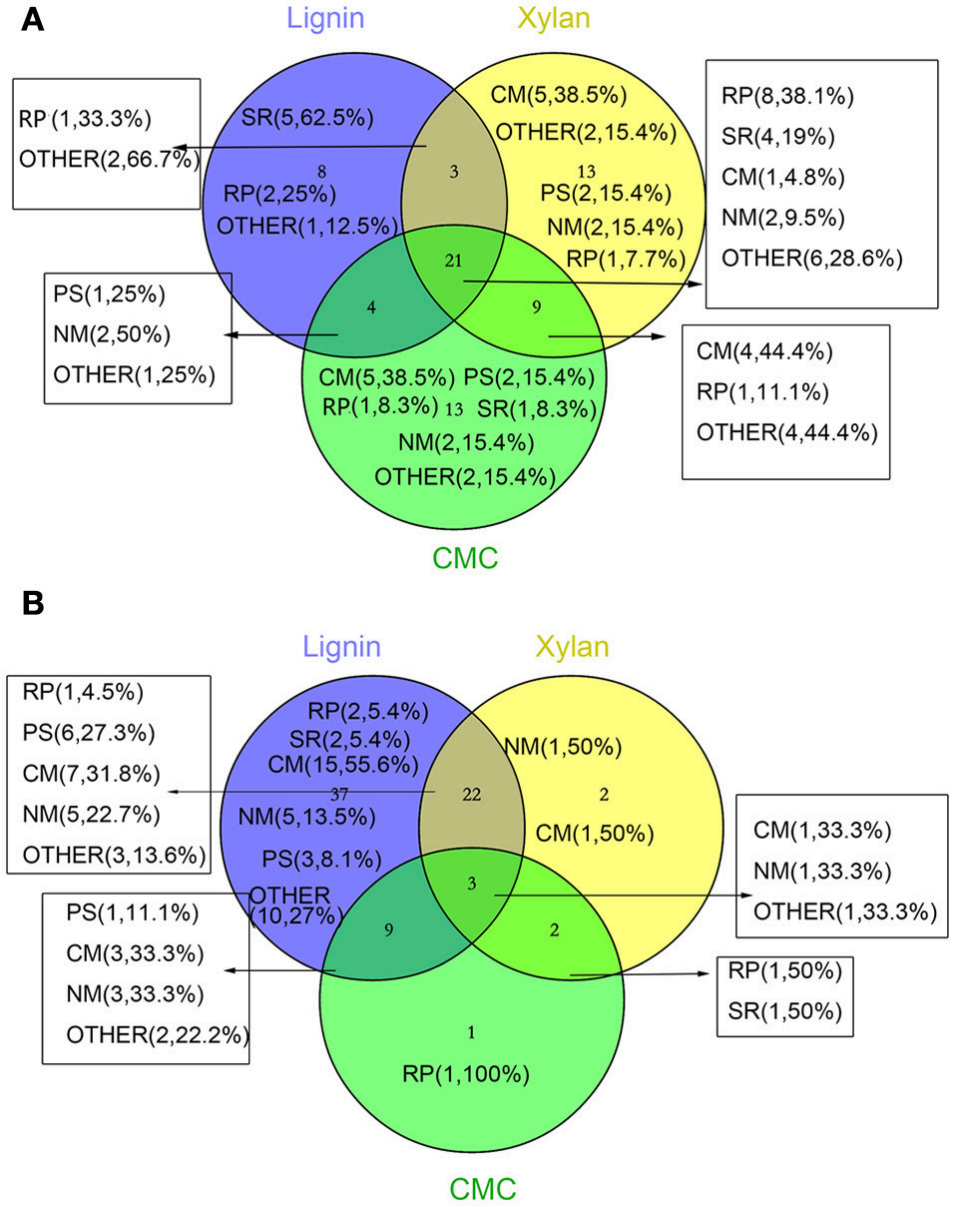
**Venn diagram representing the distribution of number and function of validated and significantly changed proteins according to proteome**. The numbers in parentheses indicate the amount and percentage (the percentage of the proteins in increased or decreased proteins in different treatments) of protein in this section. **(A)** increased proteins **(B)** decreased proteins. Abbreviations refer to different metabolic processes: RP, redox process; NM, nucleotide metabolism; PS, protein and amino acid synthesis; SR, stress response; CM, Carbohydrate metabolism and energy metabolism; OTHER, other metabolism and unkown function.

**Figure 6:**
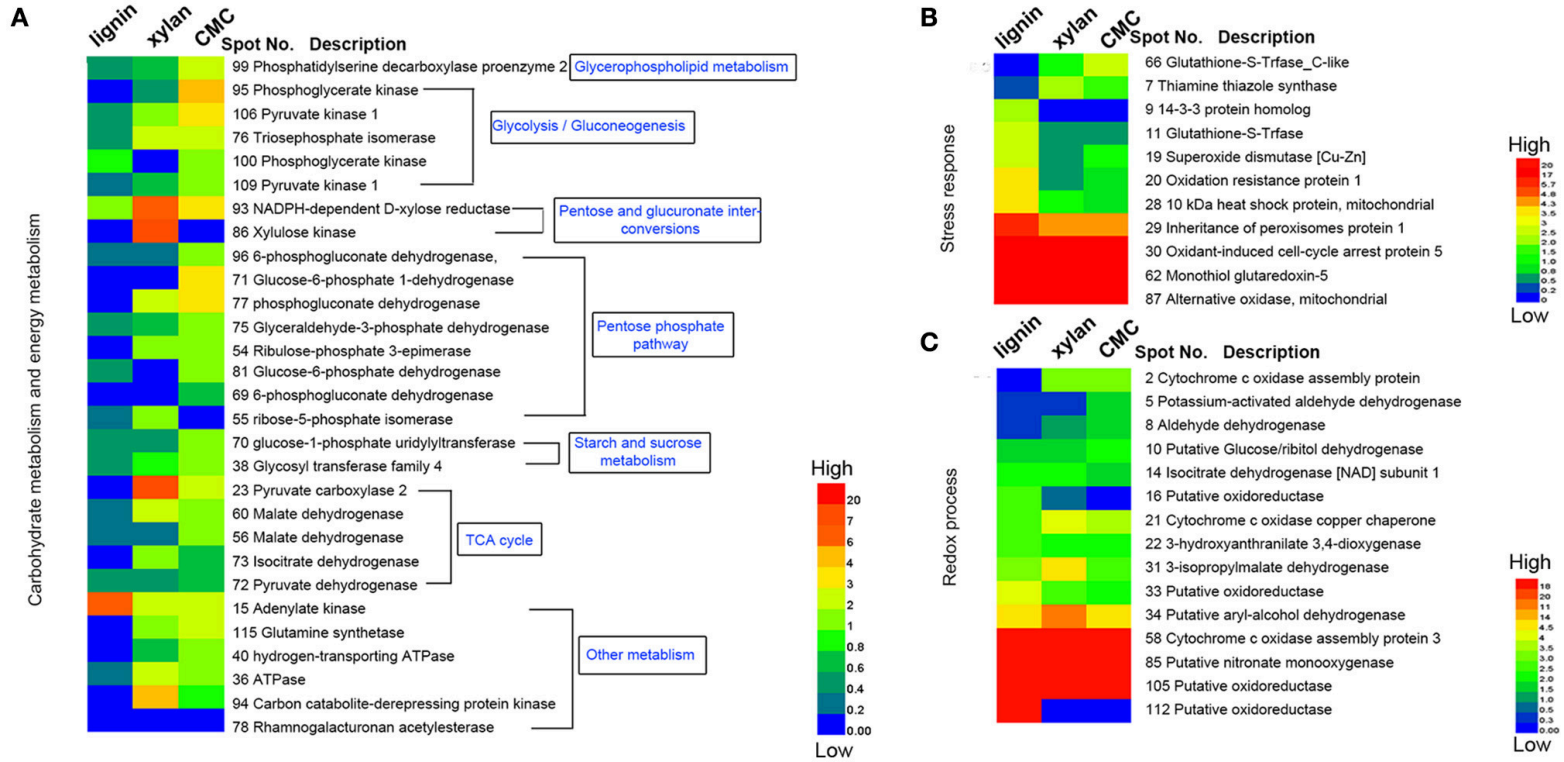
**Heatmap of the fold changes of differential proteins related to carbohydrate metabolism, stress response and redox process. (A)** Fold change of proteins related to carbohydrate metabolism and energy metabolism. **(B)** Fold change of proteins response to stress. **(C)** Fold change of proteins related to redox process. The data are presented in matrix format in which rows represent the individual proteins and the columns represent each culture. Each cell in the matrix represents the fold change of a protein at an individual substrate compared with control. The red and green colors in cells reflect low and high change fold, respectively.

Three biological replicates for each mycelial protein of *P. ostreatus*, grown in Kirk's medium and in Kirk's medium supplemented with lignin, were separated by 2-DE. Total 531 ± 23, 496 ± 19, 567 ± 38, and 601 ± 27 protein spots were detected in the control, lignin, xylan, and CMC conditions, respectively (Figure 4). Proteins that were differentially expressed under various culture conditions were divided into categories according to their molecular functions and involvement in biological processes, based on the JGI database and GO (http://geneontology.org/) classification system (Table 1, Figures 5, 6). For proteins lacking exact functional annotations in this database, we used family and domain databases (Inter Pro and Pfam) to reveal annotations of their conserved domains. Identified proteins included those involving in (i) redox processes and (ii) stress response. The stress-response group included anti-oxidation proteins and proteins involving in the response to toxic stress that are considered to play a role in the protection of cells from damage. The intensity of four spots (6, 7, 19, 20) for proteins involving in the stress response and three spots (58, 85, 112) for proteins involving in redox processes show a significant increase in all fungi grown in the three substrates relative to the control. The identified proteins also included proteins involving in (iii) carbohydrate metabolism and energy metabolism; these proteins are involving in the conversion of carbohydrates into energy to support cell processes. Figure 5 show that the intensity of 15 spots representing proteins involving in this process was significantly decreased for fungi grown on lignin, whereas five spots representative of proteins related to carbohydrate metabolism exhibited an increase for those grown on xylan and CMC. The identified proteins also included proteins involving in (iv) protein and amino acid synthesis, (v) nucleotide metabolism, and (vi) others. Proteins in the “others” group were related to other types of metabolism or considered to have unknown functions.

**Table 1:** **List of proteins identified by ESI-MS/MS from ***P. ostreatus*** growing in lignocellulose**.

**Spot no**.	**Biological process**	**JGI ID**	**Description**	**MW(kDa)/pI**	**Mascot score**	**Fold change(treat/control)**		
						**Lignin**	**Xylan**	**CMC**
3	Carbohydrate metabolism and energy metabolism	jgi|1043453	Mannose-6-phosphate isomerase	32.9/3.91	98	a	b	a
99		jgi|1036636	Phosphatidylserine decarboxylase proenzyme 2	54.4/6.19	85	0.4	0.64	2.18
109		jgi|1070334	Pyruvate kinase 1	61.6/7.47	151	0.25	0.75	1.53
100		jgi|1011623	Phosphoglycerate kinase	41.8/3.67	169	0.95	a	1.95
76		jgi|1109049	Triosephosphate isomerase	27.4/6.85	109	0.48	2.03	2.67
106		jgi|1070334	Pyruvate kinase 1	45.6/7.69	84	0.47	1.53	3.16
95		jgi|1032066	Phosphoglycerate kinase	45.0/3.44	115	a	0.49	4.25
86		jgi|1019376	Xylulose kinase	28.9/4.01	87	a	b	a
93		jgi|1102061	NADPH-dependent D-xylose reductase	34.7/6.69	99	1.22	6.41	3.1
96		jgi|1114405	6-phosphogluconate dehydrogenase, decarboxylating	62.3/3.98	75	0.3	0.37	1.54
55		jgi|1068318	Ribose-5-phosphate isomerase	31.4/6.08	90	0.34	1.59	a
69		jgi|1109004	6-phosphogluconate dehydrogenase	53.7/6.23	147	0.14	0.17	0.69
81		jgi|1108563	Glucose-6-phosphate dehydrogenase	16.4/7.89	106	0.42	a	1.14
75		jgi|1090672	Glyceraldehyde-3-phosphate dehydrogenase	34.6/6.63	135	0.44	0.72	1.51
77		jgi|1037028	Phosphogluconate dehydrogenase	26.6/7.02	126	0.11	2.62	3.55
54		jgi|1101876	Ribulose-phosphate 3-epimerase	25.9/5.5	219	0.13	1.17	1.26
38	Carbohydrate metabolism and energy metabolism	jgi|1018327	Glycosyl transferase family 4	81.8/6.11	76	0.514	0.88	1.13
71		jgi|1096444	Glucose-6-phosphate 1-dehydrogenase	58.5/6.55	140	a	0.14	3.85
70		jgi|1064981	Glucose-1-phosphate uridylyltransferase	58.7/6.26	154	0.43	0.44	1.23
72		jgi|48499	Pyruvate dehydrogenase	44.9/7.7	163	0.41	0.57	0.67
73		jgi|48714	Isocitrate dehydrogenase	47.5/5.93	209	0.13	1.11	0.72
56		jgi|1094663	Malate dehydrogenase	34.2/6.12	139	0.3	0.35	1.23
60		jgi|1075656	Malate dehydrogenase	34.1/6.09	91	0.34	2.06	1.87
23		jgi|1097340	Pyruvate carboxylase 2	18.0/4.34	87	a	7.57	2.16
78		jgi|1113799	Rhamnogalacturonan acetylesterase	25.6/6.02	181	a	a	a
94		jgi|1082594	Carbon catabolite-derepressing protein kinase	35.0/7.45	89	a	4.82	0.96
36		jgi|1053961	ATPase	100.3/5.27	239	0.23	2.58	1.15
40		jgi|1044485	Hydrogen-transporting ATPase	57.0/5.34	204	a	0.77	1.25
115		jgi|1089099	Glutamine synthetase	22.8/6.98	148	a	1.3	2.13
15		jgi|1087999	Adenylate kinase	27.7/6.77	104	6.29	2.33	2.26
13	Nucleotide metabolism	jgi|1105829	GDP-mannose transporter	23.7/4.26	92	a	b	a
88		jgi|1099408	Suppressor of kinetochore protein 1	17.4/3.63	76	a	b	a
84		jgi|47938	Alpha-1,3/1,6-mannosyltransferase ALG2	49.7/4.09	64	0.45	2.35	a
79	Nucleotide metabolism	jgi|1037108	Scavenger mRNA decapping enzyme	25.4/6.11	204	0.19	a	a
46		jgi|1054232	Ribonuclease T2	41.5/5.92	198	0.17	0.27	0.62
47		jgi|1083505	40S ribosomal protein	32.1/5.21	72	0.38	0.15	0.94
41		jgi|1056351	cysteine-type endopeptidase	55.0/5.81	88	a	a	1.19
44		jgi|1088444	GTP binding	42.0/5.11	109	a	1.31	1.23
57		jgi|1037683	Ribosomal	33.6/5.99	95	0.3	0.92	1.23
68		jgi|1054296	GTPase	93.4/6.27	136	0.28	0.22	1.4
103		jgi|1090777	Glucosamine 6-phosphate N-acetyltransferase	30.9/3.91	99	0.45	0.45	1.55
59		jgi|1095212	Endo/exonuclease	34.7/5.25	92	0.33	0.76	1.76
113		jgi|1106249	Tethering factor for nuclear proteasome sts1	24.0/8.28	88	2.23	1.28	2.16
101		jgi|1075990	cAMP-dependent protein kinase regulatory subunit	34.1/6.25	78	a	1.63	2.19
108		jgi|1101333	40S ribosomal protein S29	45.2/7.36	144	0.46	0.61	2.63
24		jgi|1066340	60S ribosomal protein L43	14.7/4.98	106	2.15	7.78	3.34
27		jgi|1047882	Dimethyl adenosine transferase	36.1/9.0	79	2.32	0.464	4.64
90		jgi|185993	60S ribosomal protein L23-B	14.3/4.26	83	b	b	b
89	Other metabolism	jgi|1114368	Calmodulin	31.3/3.57	100	a	b	a
63		jgi|52279	Formamidase	42.9/5.22	137	0.3	0.67	0.93
39		jgi|1098883	Amidase	55.7/5.09	188	0.27	a	1.12
64		jgi|1093313	Delta-aminolevulinic acid dehydratase	35.8/5.79	151	a	1.03	1.52
65		jgi|1054502	Enolase	47.1/5.55	157	0.15	1.17	1.74
66		jgi|1107810	CoA- transferase	41.8/5.93	218	0.28	1.21	1.99
97		jgi|155235	Mitochondrial import inner membrane translocase	115.6/4.7	100	a	0.44	2.26
61		jgi|1067896	Putative hydrolase	40.5/6.32	157	0.47	1.74	2.65
25		jgi|1065645	Putative Sugar transporter	57.5/3.97	83	2.31	21.19	4.62
114		jgi|1094266	Diphosphoinositol polyphosphate phosphohydrolase	20.7/4.77	82	1.76	4.91	4.86
35		jgi|1107810	Putative CoA-transferase	41.9/5.93	256	4.29	4.78	8.18
102		jgi|1101425	Sterol 3-beta-glucosyltransferase	32.6/6.24	86	b	b	b
83	Protein and amino acid synthesis	jgi|1089644	Leucine carboxyl methyltransferase	50.0/3.47	76	a	b	a
45		jgi|1076233	3-isopropylmalate dehydrogenase	40.7/5.56	182	a	0.39	0.67
48		jgi|176309	Glucosamine-6-phosphate isomerase	32.8/5.81	136	0.47	a	0.68
74		jgi|1094925	Adenosylhomocysteinase	47.3/5.93	141	a	1.3	0.85
111		jgi|1010364	SWI5-dependent HO expression protein 3	60.2/7.74	95	a	a	0.88
37		jgi|1091537	Alanine–tRNA ligase	106.3/5.52	116	a	2.54	1.21
49		jgi|1099302	Methyltransferase	31.0/5.7	206	0.37	a	1.23
67		jgi|1060865	Aspartate aminotransferase	45.4/6.54	176	0.21	0.37	1.28
107		jgi|1112899	Phenylalanine ammonia-lyase	33.4/7.08	96	a	0.75	1.59
104		jgi|1027245	E3 ubiquitin-protein ligase TOM1	31.6/4.64	102	1.21	1.18	2.17
110		jgi|1039251	Serine/threonine-protein kinase MEC1	61.4/7.65	79	2	0.53	2.57
98		jgi|48252	Glycylpeptide N-tetradecanoyltransferase	85.2/6.66	80	a	a	b
22	Redox processes	jgi|1066477	Putative oxidoreductase	22.8/3.22	72	b	a	a
10		jgi|1027050	Cytochrome c oxidase copper chaperone	13.9/4.93	74	2.55	0.51	0.26
112		jgi|1098138	Aldehyde dehydrogenase	21.9/6.37	94	0.37	0.32	1.49
34		jgi|1045076	Putative oxidoreductase	32.8/5.56	147	2.24	2.24	1.79
58		jgi|1081301	Putative Glucose/ribitol dehydrogenase	33.1/6.17	184	0.42	1.42	1.89
105		jgi|1029362	Isocitrate dehydrogenase [NAD] subunit 1	28.3/6.19	90	1.68	1.63	2.1
31		jgi|1086766	Putative oxidoreductase	26.3/5.69	146	4.24	2.97	2.24
2		jgi|19749	Cytochrome c oxidase assembly protein	31.1/3.55	108	2.98	2.19	2.32
8		jgi|1039979	3-isopropylmalate dehydrogenase	15.5/6.24	82	3.26	6.52	2.6
85		jgi|1098138	Potassium-activated aldehyde dehydrogenase	35.4/4.65	63	a	3.38	3.24
21		jgi|1015418	3-hydroxyanthranilate 3,4-dioxygenase	18.6/4.14	80	2.67	4.22	3.76
14		jgi|1030848	Putative aryl-alcohol dehydrogenase	31.4/4.95	95	9.25	11.13	4.63
33		jgi|1076970	Putative oxidoreductase	32.3/5.5	133	b	b	b
5		jgi|1015750	Cytochrome c oxidase assembly protein 3	14.6/4.65	88	a	a	a
16		jgi|1087944	Putative nitronate monooxygenase	19.2/7.34	69	b	b	b
29	Stress response	jgi|1064479	14-3-3 protein homolog	23.0/8.26	109	2.37	a	a
30		jgi|1077356	Glutathione-S-Trfase	26.9/5.77	131	2.53	0.52	0.78
9		jgi|1077250	Oxidation resistance protein 1	14.4/5.76	105	3.52	0.69	0.84
11		jgi|185767	10 kDa heat shock protein, mitochondrial	14.4/5.76	64	4.23	1.26	0.85
28		jgi|1113505	Superoxide dismutase [Cu-Zn]	28.1/9.0	100	2.6	0.52	1.38
87		jgi|1022101	Thiamine thiazole synthase	22.8/3.52	79	0.42	2.19	1.66
62		jgi|1058013	Glutathione-S-Trfase_C-like	42.1/6.0	98	a	1.38	2.56
19		jgi|1108100	Inheritance of peroxisomes protein 1	40.0/5.42	71	5.78	4.56	4.79
6		jgi|1032742	Oxidant-induced cell-cycle arrest protein 5	16.6/3.58	97	b	b	b
7		jgi|1025559	Monothiol glutaredoxin-5	13.5/4.34	100	b	b	b
20		jgi|1106868	Alternative oxidase, mitochondrial	45.4/4.61	90	b	b	b
91	Unkown function	jgi|1048439	Uncharacterized protein C6B12.14c	15.4/4.17	111	a	b	0
50		jgi|1080037	PLC-like phosphodiesterase	32.7/5.0	89	0.14	0.2	0.41
26		jgi|1022166	Purine phosphorylase	43.7/7.33	120	2.32	2.78	0.7
51		jgi|1097135	Calcium ion binding protein	25.4/5.32	194	a	0.7	1.23
43		jgi|1048986	Predicate protein	46.5/5.34	182	a	a	1.24
80		jgi|1016239	Putative protein	15.2/5.95	122	a	1.14	1.29
53		jgi|1081199	Putative protein	16.8/5.71	94	a	0.93	1.33
18		jgi|1046446	Hypothetical protein	14.7/9.0	84	5.44	1.41	1.47
1		jgi|1075945	Hypothetical protein	34.9/3.58	69	4.47	2.77	1.52
17		jgi|1012191	Hypothetical protein	15.4/7.66	99	3.26	4.08	3.98
32		jgi|1107762	Hypothetical protein	30.0/5.38	192	9.22	1.84	4.61
92		jgi|176718	Putative uncharacterized protein YKL131W	18.8/4.76	141	1.18	5.54	6.36
52		jgi|1033638	Putative protein	21.1/4.68	91	0.48	26.11	11.14
82		jgi|1048248	Bifunctional lycopene cyclase/phytoene synthase	114.5/3.34	96	a	b	b
4		jgi|166436	Hypothetical protein	18.7/3.64	98	b	b	b
12		jgi|174389	Hypothetical protein	17.1/5.76	104	b	b	b

*a, particularly low; b, particularly high*.

### Lignin-responsive proteins

Base on the result of proteomics, the intensity of 36 spots was found to be significantly increased (fold > 2) and that of 71 spots significantly decreased (fold < 0.5; Table 1, Figure 6). Eight spots only increased or detected for fungus grown on lignin, whereas the intensity of spot 9 (oxidation-resistance protein), spot 11 (10-kDa heat shock protein), spot 28 (superoxide dismutase [Cu-Zn]), spot 29 (14-3-3 protein), and spot 30 (glutathione-S-transferase), representing proteins involving in the stress response in lignin, was 3.5-, 2.5-, 2.6-, 2.4-, and 2.5-fold higher than that of the control, respectively. Among proteins related to the redox process, the intensity of spot 10 (cytochrome c oxidase copper chaperone) increased by 2.5-fold in lignin compared to that in the control, whereas spot 22 (putative oxidoreductase) was only detected for fungus grown on lignin. Notably, spot 30 and spot 62 both corresponded to glutathione-s-transferase; however, spot 62 was not detected for the lignin group, probably because subunits of the same protein would separate during the focusing process. The intensity of 26 proteins related to carbohydrate metabolism was significantly decreased for the lignin group. Most of these proteins participate in six types of carbohydrate metabolism. Interestingly, the intensity of the carbohydrate metabolism-related protein adenylate kinase (spot 15) was 6.3-fold higher than that in the control.

### Polysaccharide-responsive proteins

Xylan and cellulose, which are the main polysaccharides present in lignocellulose, are the primary carbon sources for fungi. In this study, CMC was used as a substitute for cellulose to study the effect of cellulose on *P. ostreatus*. Differentially expressed proteins displayed similar expression patterns in xylan and CMC; for both substrates, most proteins showing an increase in abundance were associated with carbohydrate metabolism. Ten carbohydrate metabolism-related proteins showed higher abundance in the two substrates than in the control. Table 1 show that the intensity of spot 95 (phosphoglycerate kinase) and spot 106 (pyruvate kinase), which represented proteins involving in the glycolysis/gluconeogenesis pathway, was 4.3- and 3.2-fold higher in the CMC group than in the control. Spot 71 (glucose-6-phosphate 1-dehydrogenase) and spot 77 (phosphogluconate dehydrogenase), which represented proteins involving in the pentose phosphate pathway, had 3.9- and 3.6-fold higher abundance in the CMC group than in the control. However, these spots showed lower abundance in the xylan group. In addition, the intensity of spot 93 (NADPH-dependent D-xylose reductase) was 6.4-fold higher in the xylan group and 3.1-fold higher in the CMC group than in the control. Spot 86, which was identified as a xylulose kinase, was only detected in the xylan group. D-xylose reductase and xylulose kinase are both involving in the pentose and glucuronate interconversion pathway. In other species, these two proteins are involving in xylan degradation and energy release. The intensity of spot 23 (pyruvate carboxylase) was 7.6-fold higher in the xylan group than in the control group, but only 2.2-fold higher in the CMC group than in the control group.

## Discussion

Lignocellulose is the main substrate used for cultivation of edible fungi. Hemicellulose and cellulose are carbon sources for fungal growth; however, another main component of lignocellulose, lignin, affects the degradation of fiber by fungi. The presence of lignin limits the access of cellulotytic enzymes to cellulose, that may influence the efficiency of enzymatic hydrolysis of cellulose and hemicellulose (Kumar et al., 2012). This effect is not observed in white-rot fungus, in which lignin is degraded by the extracellular oxidative system. However, the growth of this fungus is affected by a series of lignin derivatives; previous studies have shown that various lignin-related para-phenolic benzoic acids, para-phenolic cinnamic acids, and para-phenolic phenylpropionic acids elicit increased inhibition of growth in white-rot fungus (Buswell and Eriksson, 1994). In addition, higher concentrations of aromatic aldehydes were shown to be more toxic than the corresponding carboxylic acid (Dekker et al., 2002). These findings are consistent with those of the previous work showing that the growth of *P. ostreatus* is inhibited by lignin (Barakat et al., 2012). In the present study, although the fungus was still able to grow on lignin, the relative growth rate increased 7 days after inoculation. The rapid growth of mycelia in the control group was presumably related to the rapid consumption of nutrients. An alternative explanation for this observation is that the fungus began to adapt to the lignin-based medium. To date, little is known about the effects of lignin on mycelial growth and the stress response in fungi.

Lignin degradation is an extracellular oxidative process, and the production of H_2_O_2_ is temporally related to lignin degradation (Achyuthan et al., 2010). *P. ostreatus* has a range of extracellular enzymes that generate H_2_O_2_ for utilization by ligninolytic enzymes (Akpinar and Urek, 2014). Superoxide dismutase, ascorbate peroxidases, and glutathione reductase are key enzymes involving in reducing H_2_O_2_ in the ascorbate-glutathione cycle in cells (Yousuf et al., 2012; Choudhury et al., 2013; Yang et al., 2013). These proteins, which are induced in response to numerous environmental stresses, mediate the detoxification of reactive oxygen species. The enzymes related to the oxidative stress response were more abundant in the lignin condition, indicating a better response to H_2_O_2_ in the mycelium of *P. ostreatus* when compared to that in other culture conditions. These proteins, which are expressed in response to increased concentrations of extracellular H_2_O_2_, scavenge excess intracellular reactive oxygen species to protect cells from oxidative damage.

Inhibition of the transformation of carbon sources is another effect of oxidative stress on *P. ostreatus* (Filomeni et al., 2015). In the present study, most proteins involving in carbohydrate and energy metabolism were less abundant in the lignin group. This suggests that the inhibition of energy metabolism in response to lignin restricts mycelial growth. In the present study, as the adaptability of fungi to lignin increased, this restriction was gradually lifted, allowing slow accumulation of mycelial biomass to occur.

Recent research has suggested that laccase may play an important role in the fungal defense against oxidative stress, which acts as an element of the stress response (Giardina et al., 2010). It has been observed that oxidative stress induces the expression of ligninolytic enzymes in some basidiomycetes (Viswanath et al., 2014). In our study, the activity of laccase increased with time in the lignin group, and the increase in laccase expression appeared to increase the resistance of *P. ostreatus* to oxidative stress. The increase in laccase activity was therefore considered to enhance the adaptability of *P. ostreatus* to lignin in a gradual manner.

Interestingly, we found that the intensity of a 14-3-3 protein was significantly increased in the lignin group. The 14-3-3 proteins, which are rarely reported in fungi, are known to be upregulated in plants in response to pathogenic fungi. Previous studies have suggested that 14-3-3 proteins may control a negative feedback loop to prevent harmful overactivation of defense responses in plants (Lozano-Durán and Robatzek, 2015). Our results suggest a prominent role for 14-3-3 proteins in the fungal response to stress; however, it is not clear how lignin regulates the expression of this protein. The question of whether the expression of this protein relates to lignin needs further study.

The present results elucidate the relationship of the expression of antioxidative intracellular proteins and laccase with the defense response to exogenous H_2_O_2_—induced oxidative stress in fungi grown on lignin (Strong and Claus, 2011). Although the expression of these proteins promoted the adaptability of *P. ostreatus* to lignin, it is possible that alternative stress response mechanisms may additionally be associated with adaptation to growth in such environments.

Cellulose and hemicellulose in lignocellulose are the main nutrient sources for *P. ostreatus*. In fungi, the cAMP–PKA and TOR pathways respond to carbon and nitrogen signals to regulate a myriad of functions, including protein synthesis, ribosome biogenesis, autophagy, polarized cellular growth, cell-cycle progression, and filamentation (Liu et al., 1993). TOR signaling activates the expression of genes required for ribosome biogenesis, including those encoding ribosomal proteins, ribosomal RNA (rRNA), and tRNA (Dobrenel et al., 2016). Our findings additionally showed that cAMP-dependent protein kinase and three ribosomal proteins involving in sugar sensing were significantly upregulated in fungi grown on xylan and CMC. Furthermore, xylan and CMC regulate the adaptation of the fungus to the environment via their signaling pathways. Therefore, after inoculation for 7 days, the mycelial growth rate was observed to increase rapidly.

In a previous study, sensing of glucose as the preferred carbohydrate source was extensively studied in the yeast model organism (Braunsdorf et al., 2016). In the presence of glucose, genes required for growth on alternative carbon sources are repressed (Bahn et al., 2007). For *P. ostreatus*, the natural growth environment lacks glucose; accordingly, this fungus has evolved an effective method for regulation of natural polysaccharides. Various filamentous fungi, including *Neurospora crassa*, are capable of growth on pentose (Li et al., 2014). The genomes of pentose-utilizing fungi are a useful resource for mining novel gene elements, such as D-xylose transporters for metabolic engineering in *S. cerevisiae*. The xylose metabolism pathway consists of three enzymes, namely xylose reductase, xylitol dehydrogenase, and xylulokinase, which have been studied in relation to the metabolic engineering of *S. cerevisiae* for xylose fermentation (Farwick et al., 2014). This has been a subject of great interest over the past decade, as xylose is easier to obtain in nature (Li et al., 2015a). Despite these endeavors to improve xylose fermentation, the yields and productivity for ethanol obtained from xylose, using engineered *S. cerevisiae*, are much lower than those for ethanol obtained by glucose fermentation (Kurosawa et al., 2013). The high intensity of D-xylose reductase and xylulose kinase in *P. ostreatus* grown on xylan may be related to increased xylose metabolism under xylan regulation. However, this is not the only carbon metabolism pathway that is enhanced under xylan regulation; the expression of malate dehydrogenase, pyruvate carboxylase, ATPase, and adenylate kinase, which are involving in TCA metabolism, is also increased on xylan. The enhancement of xylose metabolism and other carbohydrate metabolism pathways greatly promotes the utilization of polysaccharides by *P. ostreatus*.

The hydrolysis product of CMC is glucose; therefore, the response mechanism of *P. ostreatus* for CMC is similar to that for glucose. Previous studies proved that GTPase activity may be indicative of the activation of signaling pathways in the presence of glucose as a carbon source, and almost half of the identified signaling-related proteins are G-protein coupled receptors or small GTPases (Post and Brown, 1996; Gancedo, 2008). GTPases are present at high levels in CMC, suggesting that it activates this signaling pathway. Addition of glucose to cells growing on non-fermentable carbon sources, or to stationary-phase cells, triggers a wide variety of regulatory processes directed toward the exclusive and optimal utilization of the preferred carbon source (Gancedo, 2008). Pyruvate kinase, phosphoglycerate kinase, triosephosphate isomerase, and phosphoglycerate kinase are upregulated in fungi growing on CMC, suggesting that glycolysis is activated by glucose. When glucose influx and utilization through glycolysis are stimulated, gluconeogenesis is inhibited, and there is a drastic increase in growth rate, which is preceded by a characteristic upshift in ribosomal RNA and protein synthesis.

Sugars such as xylan and cellulose are the primary fuel for most fungi (de Souza et al., 2014). The amount of available sugar may fluctuate widely, necessitating a mechanism for sensing available amounts and responding appropriately. In most organisms, this response involves changes in gene expression. Studies of the yeast glucose repression system have provided novel insights into the signaling pathway that responds to sugar. When yeast cells growing on high levels of sugar obtain most of their energy via fermentation, large amounts of sugar are metabolized through glycolysis (Johnston, 1999; Kim et al., 2013). Our findings suggest that addition of CMC and xylan to the medium significantly enhances the ability of *P. ostreatus* to transform sugars via different metabolic pathways, and improves the adaptability of *P. ostreatus* to the environment.

Alcohol oxidation is critical for lignocellulose degradation. In our study, aryl-alcohol dehydrogenase enzymes showed higher abundance in all of the lignocellulose substrates. Moreover, aryl-alcohol dehydrogenase coupled with NADPH as a co-factor constitutes a redox system involving in aryl-alcohol/aryl-aldehyde production in the fungus that ensures steady availability of H_2_O_2_ for ligninolytic activities (Yang et al., 2012). Recent studies have shown that aryl-alcohol oxidases and dehydrogenase are induced by lignin derivatives and are involving in their metabolism *in vitro* (Feldman et al., 2015). Our results suggest that aryl-alcohol dehydrogenase is induced by lignin as well as lignocellulosic polysaccharides, and regulated by lignocellulose.

Flavin-containing oxidases catalyze a wide variety of different oxidation reactions; in the last decade, many flavoprotein oxidases with varied substrate specificities and reactivities have been discovered (Dijkman et al., 2013). Glucose oxidase, the best-known flavoprotein, is involving in lignocellulose degradation (Hernández-Ortega et al., 2012). To date, few studies have focused on the correlation between flavoprotein and lignocellulose degradation in cells. The only flavoproteins known to be involving in this process are the flavin-containing monooxygenases, which are widely distributed within living organisms and involving in various biological processes such as the detoxification of drugs, biodegradation of environmental aromatic compounds, and biosynthesis of antibiotics (Nakamura et al., 2012). In our study, the level of FAD increased with time; the level of FAD in fungus grown on lignocellulose was higher than that in fungus grown in the control medium, and highest in fungus grown on lignin. This indicates that the expression of FAD is regulated by lignocellulose, and that flavoprotein in cells plays an important role in the response to lignocellulose. Although it was not possible to determine which proteins are specifically regulated by lignocellulose, our findings provide novel insights into the roles of intracellular flavoproteins in the response to lignocellulose.

Some studies have shown that *P. ostreatus* selectively degrades hemicellulose when cultured with solid biomass (Ander and Eriksson, 1977; Chandra et al., 2007). This implies that *P. ostreatus* favors the use of hemicellulose as a carbon source. In our study, xylan had a certain effect on the accumulation of mycelial biomass, and we believe that xylan plays a key role in the regulation of genes related to the metabolism of xylulose. We speculate that the selective degradation of hemicellulose when *P. ostreatus* is cultured in solid biomass occurs because xylan is a carbon source that is beneficial for the growth of *P. ostreatus* (Dwivedi et al., 2011), and xylan activates the expression of genes in the xylose-related metabolic pathway, which allows *P. ostreatus* to use hemicellulose as a carbon source. There are some reports that lignin in natural lignocellulose limits the growth of fungi, that because of the structural limitation of mycelial invasion and the use of other polysaccharides (Sattler and Funnell-Harris, 2013). Our results suggest that this restriction may also be due to the inhibition of mycelial growth by lignin and the effect of lignin on carbon metabolism in *P. ostreatus* hyphae. Our results provide further understanding of the solid-state culture of *P. ostreatus*.

Elucidation of lignocellulose–fungal interactions is important for understanding fungal ecology and for the maintenance of the delicate balance of fungal symbionts in our ecosystem. Understanding the mechanism of the fungal response to lignocellulose will facilitate its application in metabolic engineering of biotechnology to optimize the bioconversion of biomass resources in the future.

## Author contributions

XZ and CL designed the experiments. QX, FM, and HY wrote the manuscript. QX conducted most of the experimental work and performed analysis of data. YL assisted with experiments. All authors discussed the results and reviewed the manuscript.

## Funding

This research was supported by the National Basic Research Program of China (2014CB138303), the High-tech Research and Development Program of China (2012AA101805) and the National Natural Science Foundation of China (J1103514).

### Conflict of interest statement

The authors declare that the research was conducted in the absence of any commercial or financial relationships that could be construed as a potential conflict of interest.
